# A Pedunculated Skin Lesion in a Case of Clear Cell Renal Carcinoma

**DOI:** 10.7759/cureus.5021

**Published:** 2019-06-27

**Authors:** Meghana Kesireddy, Amit Correa, Raoul Correa, Rohit Venkatesan

**Affiliations:** 1 Internal Medicine, The University of Texas Medical Branch, Galveston, USA; 2 Oncology, The University of Texas Medical Branch, Galveston, USA; 3 Internal Medicine, West Virginia University, Morgantown, USA

**Keywords:** renal carcinoma, cutaneous, metastasis, treatment

## Abstract

Clear cell type renal carcinoma accounts for about 80% of all renal cell carcinomas. We present a 39-year-old male with clear cell renal carcinoma of the right kidney, stage I: T1 b (5 cm) N0 M0, who developed cutaneous metastases in the right submandibular region 28 months after nephrectomy. Our case is unique as i) the patient with stage I cancer (at the time of nephrectomy) presented with an isolated cutaneous nodule in a location distant from the primary site; ii) cutaneous nodule developed while being treated with pazopanib for metastatic lesions in the lung and adrenal; and iii) nivolumab and ipilimumab combination therapy decreased the vascularity of the nodule though it did not halt the nodule growth. Physicians should be knowledgeable about this rare clinical entity and its varied presentation. Further studies are necessary to determine optimal treatment, as the current therapeutic agents for metastatic renal carcinoma might not be adequate for cutaneous metastasis.

## Introduction

Clear cell type renal cell carcinoma accounts for about 80% of renal cell carcinomas. This is only about 2% of all adult carcinomas [1]. In renal cell carcinoma, metastasis can occur at the time of diagnosis or at any time after nephrectomy. Common sites of metastasis are lung, bone, liver, brain, and adrenal [2-3]. Cutaneous metastases in renal cell carcinoma are extremely rare, seen only in 1%-3% of the cases, and are associated with worse prognosis [3]. We present a clear cell renal carcinoma patient with metastasis to the skin of the right submandibular region; both the location of the metastasis and the presentation are unusual.

## Case presentation

**Figure 1:**
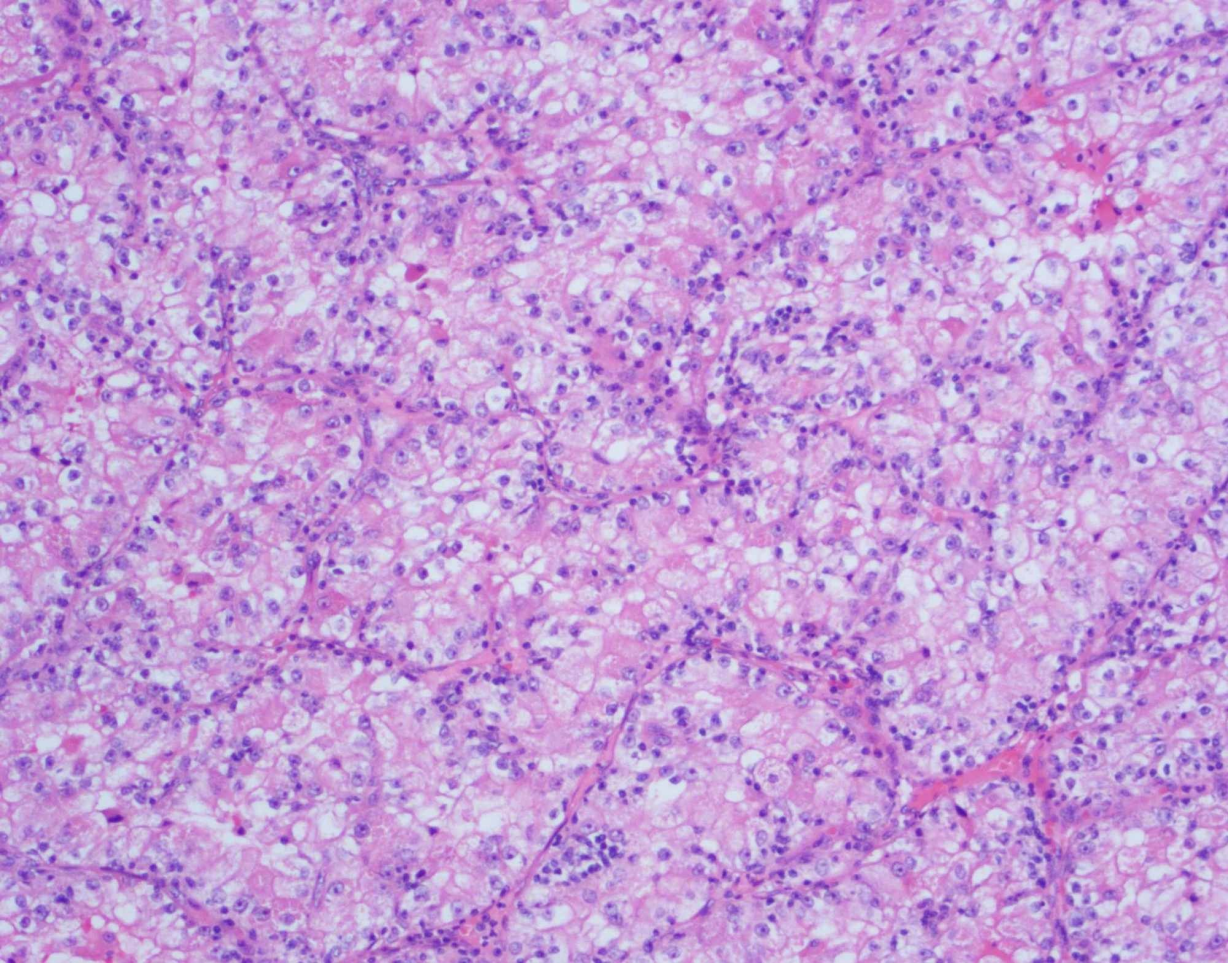
Eosin and hematoxylin stain of clear cell carcinoma of the right kidney with 100X magnification. The tumor cells have abundant clear (lipid-rich) cytoplasm with a prominent cell membrane.

**Figure 2:**
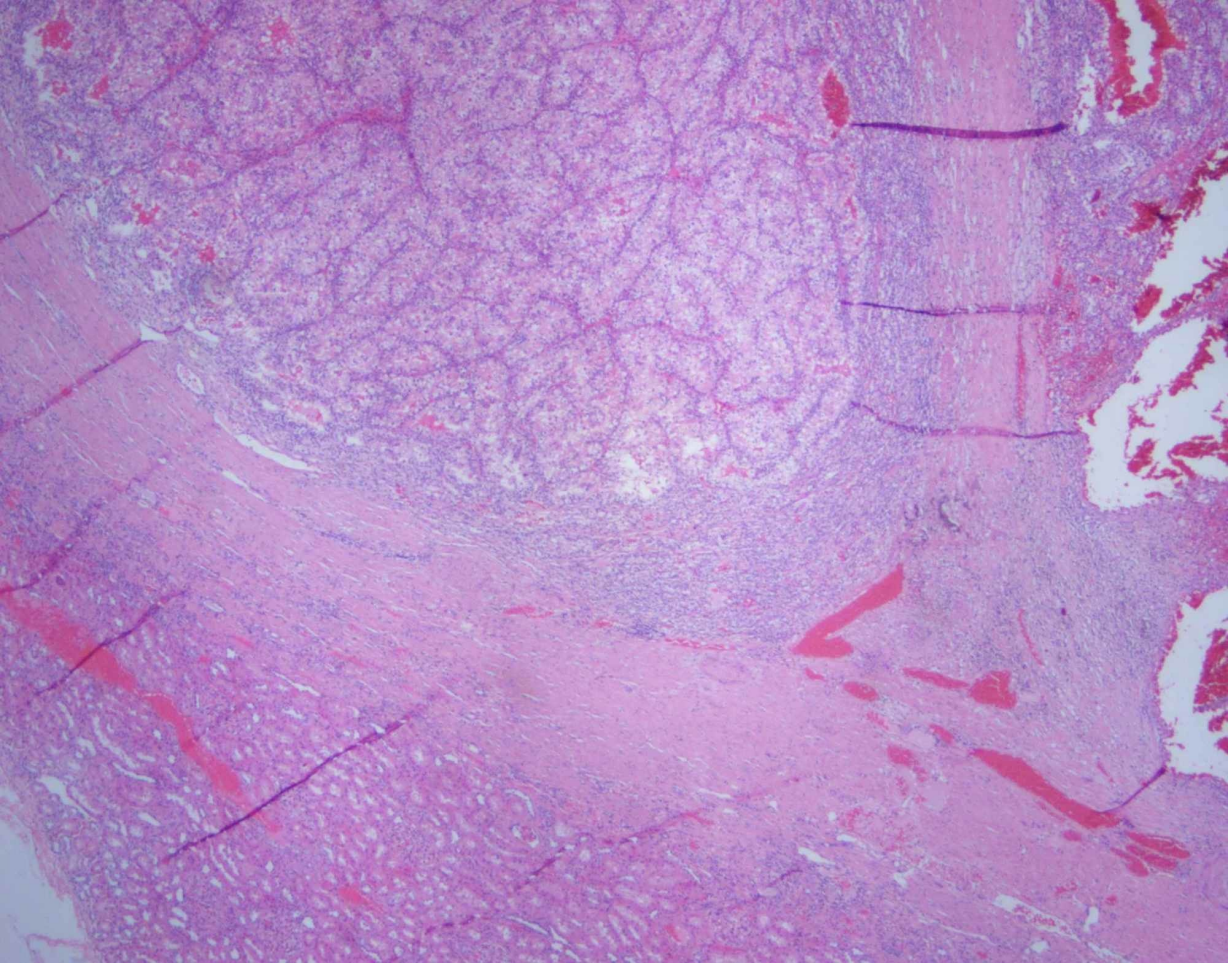
Eosin and hematoxylin stain of clear cell carcinoma of the right kidney with 20X magnification. The tumor cells are arranged in a trabecular pattern.

A 39-year-old African American male was diagnosed with renal cell carcinoma of the right kidney, clear cell type, Fuhrman grade 3, TNM stage I: T1b N0 M0, and underwent partial nephrectomy with clear margins (Figures 1-2).

He was followed with serial computed tomography (CT) thorax, abdomen and pelvis, every three months. Fourteen months later, his CT thorax revealed multiple sub-centimeter bilateral lung nodules that progressively increased in size over the next few months. Endobronchial ultrasound (EBUS)-guided biopsy of the lung nodule confirmed metastatic clear cell renal carcinoma. He was treated with different anti-angiogenic and immunotherapy agents for the next 14 months as follows. He was initially started on nivolumab (anti-programmed cell death protein 1 antibody) once every two weeks; however, a follow-up CT thorax obtained after five months showed a progression of the lung nodules. He was switched to sunitinib (vascular endothelial growth factor tyrosine kinase inhibitor) every day but could not tolerate it for more than a month due to severe diarrhea and nausea. He was then switched to temsirolimus (mammalian target of rapamycin inhibitor) once every month, and a follow-up CT thorax and abdomen obtained after four months showed a progression of the lung nodules along with the development of new adrenal nodules and right kidney mass. So, he was switched to pazopanib (vascular endothelial growth factor tyrosine kinase inhibitor) every day.

**Figure 3:**
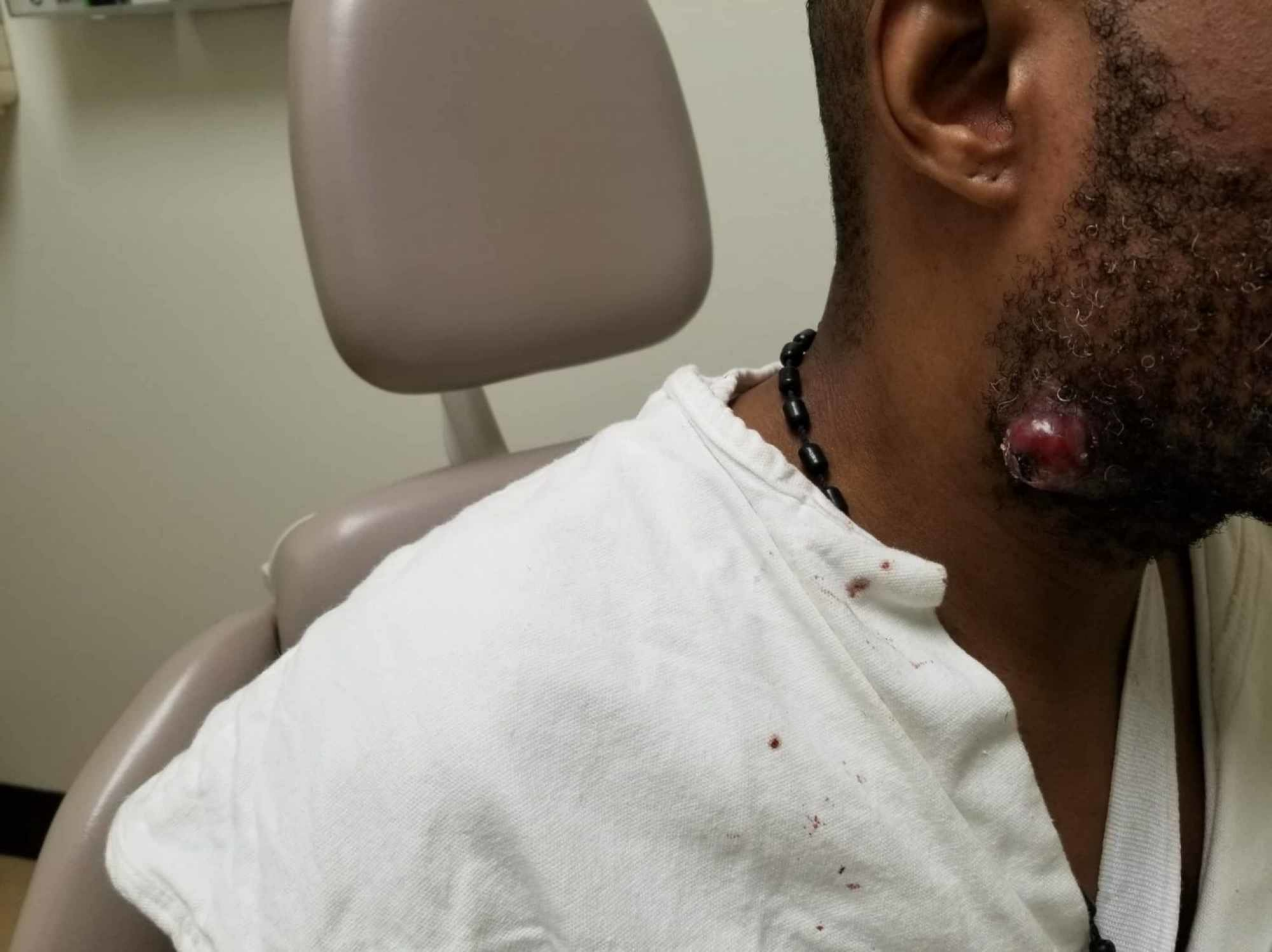
Metastatic nodule at the right submandibular region resembling a hemangioma

Three months later, CT thorax showed that metastatic lung and adrenal nodules decreased in size with pazopanib. However, during the fourth month of pazopanib therapy, he developed a small, painless papule (pimple-like lesion) over the right submandibular region. Over the next six weeks, this pimple-like lesion rapidly grew into a pedunculated, highly vascular, 1 X 1 cm (clinical size) nodule with a prominent punctum and constant serosanguinous discharge (Figure 3).

**Figure 4:**
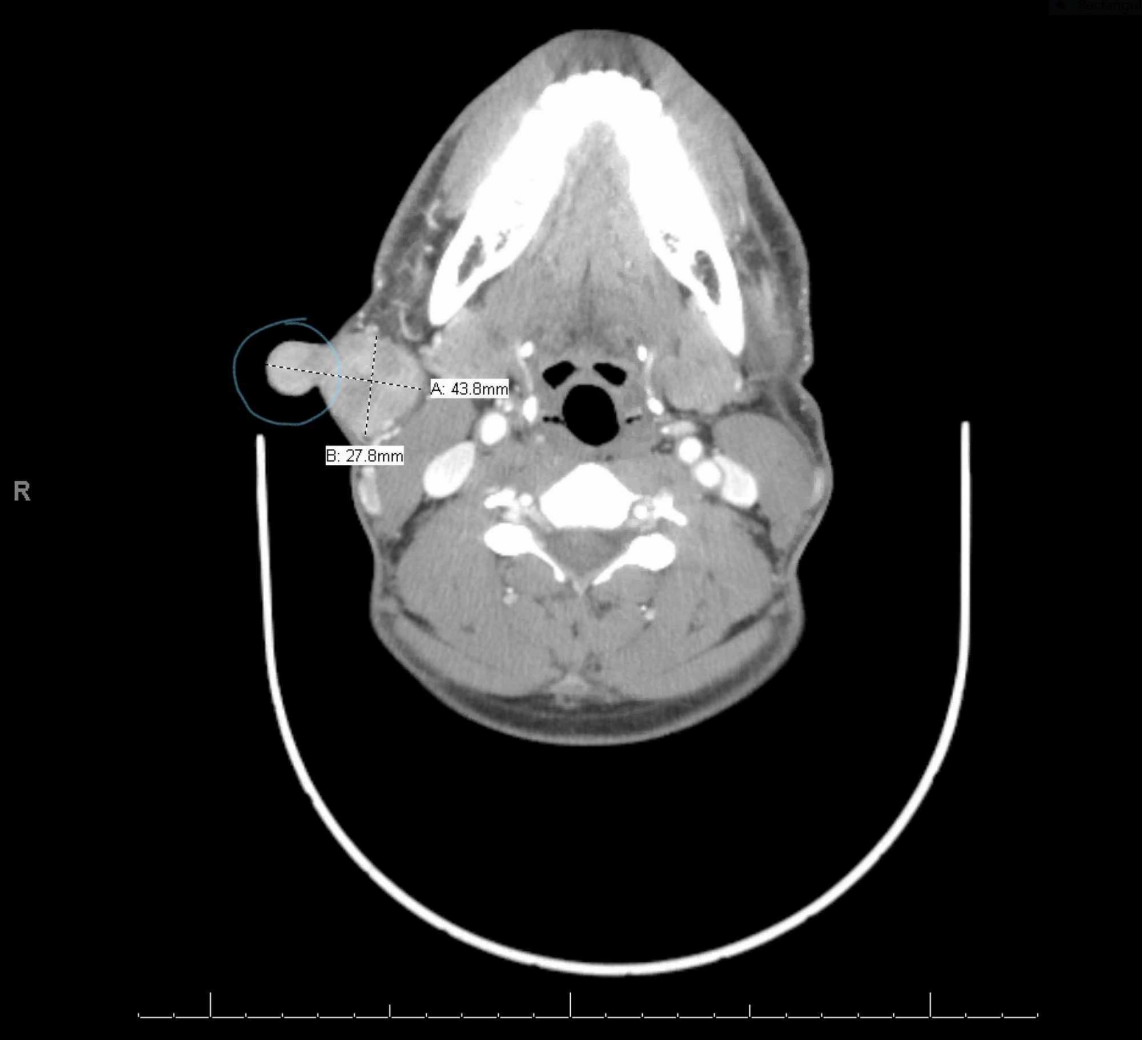
CT neck with contrast showing an avidly and heterogeneously enhancing right neck mass measuring 4.38 cm X 2.78 cm X 3 cm, at the level of the hyoid bone, superficial to the platysma with no overt invasion through the platysma. The blue circle drawn on the nodule is the externally (clinically) visible part measuring 1 X 1 cm (at the time of the computed tomography (CT) scan).

Though the clinical (external) size of the nodule was only 1 X 1 cm, CT of the neck showed a much larger, right-sided, bi-lobed heterogeneously enhancing nodule measuring 4.38 x 2.78 x 3 cm, superficial to the platysma, and at the level of the hyoid bone (Figure 4).

**Figure 5:**
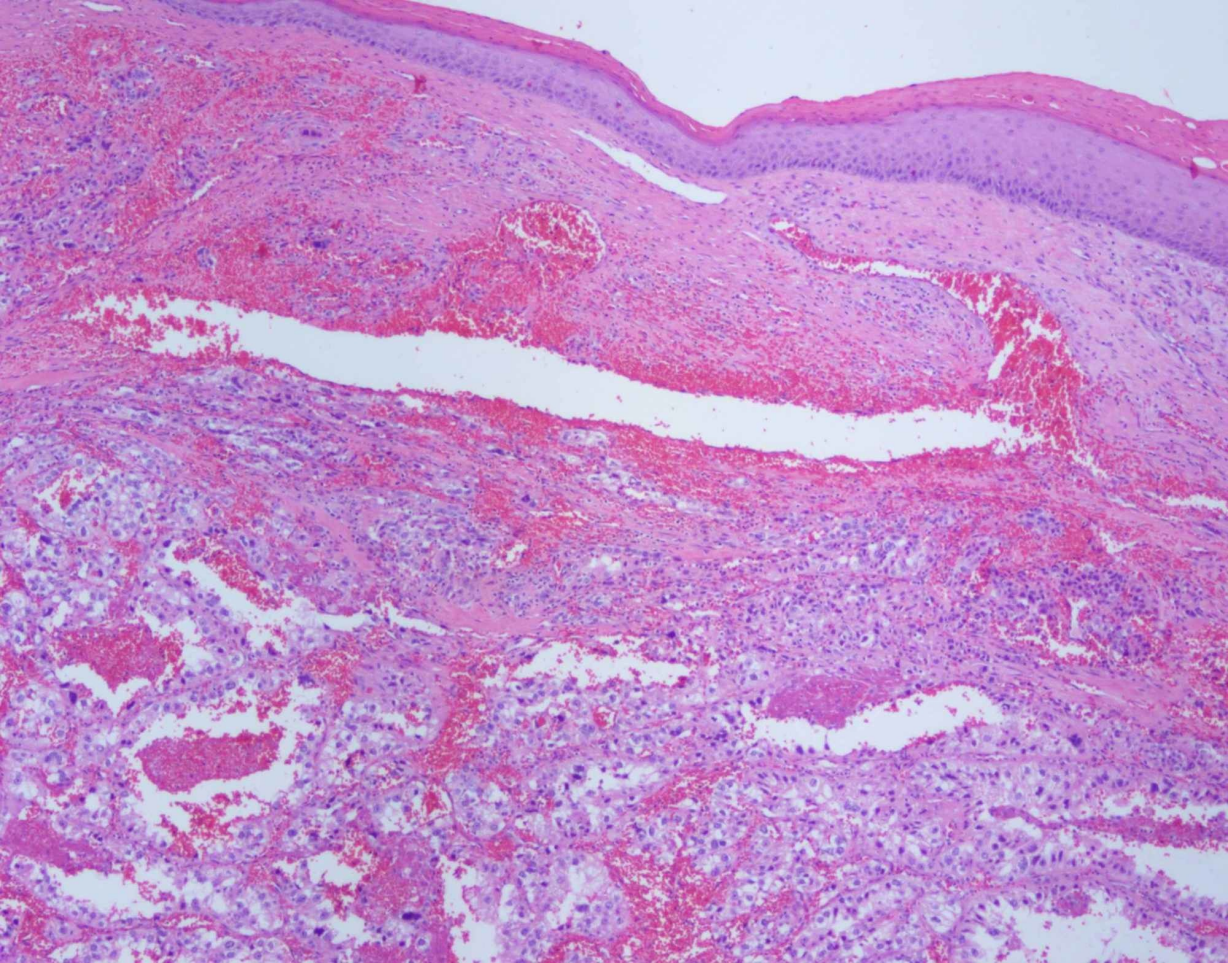
Eosin and hematoxylin stain of the metastatic cutaneous nodule with 40X magnification. Atypical, clear cells with abundant cytoplasm arranged in a trabecular pattern consistent with metastatic clear cell renal carcinoma.

Fine-needle aspiration (FNA) of the nodule showed a cohesive group of atypical cells, with ill-defined edges, abundantly wispy cytoplasm, cytoplasmic vacuoles, eccentrically located nucleoli, and arranged in a trabecular pattern. Immunohistochemistry was positive for Pax 8- consistent with metastatic clear cell renal carcinoma (Figure 5).

Pazopanib was discontinued and was started on nivolumab plus ipilimumab combination therapy once every three weeks. The cutaneous nodule continued to grow and evolved into a 3 X 3 cm (clinical size) pedunculated mass, but the vascularity of the nodule decreased as evidenced by reduced bleeding (serosanguinous discharge) with the nivolumab and ipilimumab combination therapy. One month later, he underwent surgical excision of the cutaneous mass.

## Discussion

Cutaneous metastases in renal cell carcinoma are extremely rare, seen only in 1%-3% of the cases. The incidence of cutaneous metastases is even lower in patients with stage I cancer (staging at the time of nephrectomy) as compared to stage III or higher. The average time for the onset of cutaneous metastases is longer in stage I, which is 51 months as compared to stage III or higher, which is 13 months [4]. Our patient was stage I (at the time of nephrectomy) and presented with cutaneous metastases 28 months after nephrectomy, which is earlier as compared to the reported mean time of onset. Based on our literature search, this is the only reported case where the patient developed cutaneous metastases when the visceral metastatic lesions (i.e., in the lung and adrenal) were being treated and responding to an anti-angiogenic agent (pazopanib). This case signifies that cutaneous metastases can occur with any cancer stage (staging at the time of nephrectomy) and at any time.

The common sites of cutaneous metastases are chest and abdomen due to anatomic proximity to the kidneys and scalp due to lymphohematogenous spread [4-6]. Our patient presented with an isolated lesion at the right submandibular area. which is very distant from the primary site due to lymphohematogenous spread. The metastatic cutaneous lesions in renal cell carcinoma have a wide range of clinical presentation, in terms of color and shape, and can be easily mistaken for benign skin lesions like comedones, hemangioma, pyogenic granuloma, sebaceous cyst, and more. In our patient, on clinical examination, the cutaneous lesion appeared like a comedone during initial presentation and resembled a hemangioma during later stages as it continued to grow. FNA or biopsy of the cutaneous lesion with a histopathological examination is necessary for making a diagnosis of cutaneous metastases. This case signifies that it is critical for physicians to have a high degree of suspicion for cutaneous metastases and perform a thorough physical examination while evaluating patients with a history of renal cell carcinoma.

Cutaneous metastases in renal carcinoma are associated with a worse prognosis. The mean duration of survival after the diagnosis of cutaneous metastasis is around 12 months [3-4], but there have been case reports of patients who survived for 72 months despite having disseminated renal carcinoma in other visceral sites, implying that prompt diagnosis and treatment is critical [7]. Treatment for cutaneous metastases depends on the extent of metastases. For a single, isolated cutaneous lesion, surgical removal is the treatment of choice and radiotherapy is an alternative in non-surgical patients [8]. However, most patients with cutaneous metastases have synchronous metastases in other sites and are treated with systemic therapy +/- surgical removal. Anti-angiogenic, multikinase inhibitor agents like sunitinib or pazopanib (vascular endothelial growth factor tyrosine kinase inhibitors), which are the first-line agents for metastatic renal carcinoma are commonly used [4,9]. In our patient, though pazopanib decreased the size of the visceral metastatic (lung and adrenal) nodules, cutaneous metastases developed at the same time, implying that the anti-angiogenic, multikinase inhibitors might not be an effective therapy for cutaneous metastases. Nivolumab and ipilimumab combination therapy (immune checkpoint inhibitors), an alternative first-line agent approved for metastatic renal carcinoma in 2018, decreased the vascularity of the cutaneous nodule but not its size or growth [10]. This case signifies that current therapeutic agents for metastatic renal carcinoma might not be adequate for the cutaneous metastasis.

## Conclusions

Cutaneous metastasis in renal carcinoma is associated with a worse prognosis and survival depends on prompt diagnosis and treatment. Physicians should be knowledgeable about this rare clinical entity and its varied presentation. Further studies are necessary to determine the optimal treatment for metastatic renal carcinoma in the setting of cutaneous metastasis.
